# Tunable Synthesis of SiC/SiO_2_ Heterojunctions via Temperature Modulation

**DOI:** 10.3390/ma11050766

**Published:** 2018-05-10

**Authors:** Wei Li, Quanli Jia, Daoyuan Yang, Xinhong Liu

**Affiliations:** 1 Henan Key Laboratory of High Temperature Functional Ceramics, Zhengzhou University, 75 Daxue Road, Zhengzhou 450052, China; weilizzu@hotmail.com; 2 School of Materials Science and Engineering, Zhengzhou University, 100 Kexue Road, Zhengzhou 450000, China; yangdaoyuan@zzu.edu.cn

**Keywords:** SiC/SiO_2_ heterojunctions, chemical vapor reaction, fiber, molten salt-mediated, photoluminescence

## Abstract

A large-scale production of necklace-like SiC/SiO_2_ heterojunctions was obtained by a molten salt-mediated chemical vapor reaction technique without a metallic catalyst or flowing gas. The effect of the firing temperature on the evolution of the phase composition, microstructure, and morphology of the SiC/SiO_2_ heterojunctions was studied. The necklace-like SiC/SiO_2_ nanochains, several centimeters in length, were composed of SiC/SiO_2_ core-shell chains and amorphous SiO_2_ beans. The morphologies of the as-prepared products could be tuned by adjusting the firing temperature. In fact, the diameter of the SiO_2_ beans decreased, whereas the diameter of the SiC fibers and the thickness of the SiO_2_ shell increased as the temperature increased. The growth mechanism of the necklace-like structure was controlled by the vapor-solid growth procedure and the modulation procedure via a molten salt-mediated chemical vapor reaction process.

## 1. Introduction

One-dimension (1D) SiC heterojunctions have attracted a considerable attention for broad applications in high-frequency, high-temperature nanoelectronics, nanophotonics, etc., because of their excellent properties, including higher mechanical strength, higher thermal stability, good corrosion resistance, good electrical and optical properties [1,2]. Among these, 1D SiC/SiOx nanochain heterojunctions, which combine the unique properties of SiC nanowires (NWs) and SiOx beans in the SiC/SiO_2_ core-shell structure, possess excellent optical and electrical properties, suggesting promising future applications in emitting devices [3].

To date, many methods have been exploited to synthesize 1D SiC/SiOx heterostructures, including carbothermal reduction [4], pyrolysis of a polymer precursor [5], chemical vapor deposition [6], chemical vapor reaction (CVR) [7,8], thermal evaporation [9], microwaves [10], arc discharge [11], high-frequency induction heating [12], etc. For instance, Kim et al. [13] synthesized SiC/SiO_2_ nanocables on a Ni(NO_3_)_2_-catalyzed Si substrate using thermal decomposition of methanol. Hou et al. [14] performed a large-scale production of SiC/SiOx nanochain heterojunctions via pyrolysis of polymeric precursors, the as-obtained highly purity heterojunctions comprised of single-crystalline 3C–SiC string cores and amorphous periodic SiOx bead shells. Liu et al. [9] obtained SiC/SiOx nanochain heterojunctions via thermal evaporation at 1500 °C using silicon wafer as a precursor. Meng et al. [15] prepared SiC/SiO_2_ core-shell NWs using Si, SiO_2_, and C_3_H_6_ as raw materials via a CVR method with an iron catalyst. Liu et al. [16] fabricated dumbbell-shaped biomimetic SiC/SiO_2_ fibers using gangue and carbon black as raw materials via a carbothermal reduction technique at 1500 °C, and the size of the SiO_2_ beads could be tailored by adjusting the holding time at 1300 °C in the cooling process. Unfortunately, those methods suffered from various disadvantages, such as the use of expensive raw materials, the requirement of high reaction temperatures, the low-yield production, and using of protective or reactive gas. Industrially, it is still the main bottleneck to obtain a mass production of SiC/SiOx nanochain heterojunctions.

We have successfully prepared ultra-long necklace-like SiC/SiO_2_ heterojunctions without metallic catalyst or flowing gas at 1200 °C, via a molten-salt-mediated, simplified CVR technique [17]. The morphology of the SiC/SiOx heterojunctions was that of neck-like nanochains, and the diameter of the SiOx bean was 2–6 μm. As reported in previous studies, the size of the SiOx bean was about 50 nm after firing at 1500 °C [9,14,16]. Why were the morphologies of the SiC/SiOx heterojunctions so different after firing at a different temperature? In this work, the effect of the firing temperature on the morphological evolution of SiC/SiO_2_ heterojunctions was further studied in the attempt to answer this question. We found that the microstructure of the as-prepared products could be designed by adjusting the firing temperature, indicating that the diameter of the SiO_2_ beans decreased, whereas the diameter of the NWs and the thickness of the shell increased. Moreover, their phase composition and photoluminescence properties were identified, and a growth mechanism was proposed.

## 2. Materials and Methods

A typical synthesis procedure of SiC/SiO_2_ heterojunctions was carried out in an ordinary furnace, using the experimental apparatus shown in our previous paper [17]. Silicon powder (purity 99.0 wt %, D_50_ = 27 µm) was used as a silicon source. NaCl (purity 99.0 wt %) and NaF (purity 99.0 wt %) were selected as molten salts. Firstly, mixtures of silicon powder and molten salts were placed in a corundum crucible covered with a corundum lid, and the crucible was embedded in graphite powder. Then, the mixtures were heated from room temperature to 1100 °C, 1200 °C, 1300 °C, and 1400 °C for 2 h in air atmosphere. After cooling the furnace to room temperature, the cotton-like products in the corundum crucible were collected.

X-ray diffraction (XRD, X’pert, Philips, Amsterdam, The Netherlands) with CuKα radiation was adopted to determine the crystal phase of the specimens at 40 kV and 40 mA. The chemical bond was certified by Fourier-transform-infrared spectroscopy (FT-IR) (Nicolet, IS50, Thermo Scientific, Waltham, MA, USA) in the wavenumber range of 350–1200 cm^−1^. The microstructure and morphologies of the as-synthesized products were characterized by a scanning electron microscope (Zeiss, EVO HD15, San Diego, CA, USA) equipped with an energy dispersive X-ray spectroscope (EDS, Oxford, XMAX20, Milpitas, CA, USA), along with a high-resolution transmission electron microscope (TEM–HRTEM, JEOL, JEM-2100, Tokyo, Japan). Photoluminescence (PL) spectra at room temperature were recorded by fluorescence spectrophotometery (Fluorolog, Horiba, Jobin Yvon, Madison, WI, USA), with Xe lamp excitation.

## 3. Results and Discussion

### 3.1. Morphology

**Figure 1:**
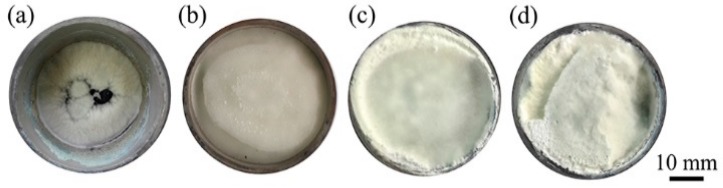
Digital image of the as-achieved products: (**a**) 1100 °C; (**b**) 1200 °C; (**c**) 1300 °C; (**d**) 1400 °C.

Figure 1 shows the digital photos of the as-synthesized products grown in the corundum crucible. As shown in Figure 1a, only a small amount of products was obtained at 1100 °C but, when increasing the temperature to 1200 °C, large quantities of cotton-like products formed in the corundum crucible, and a mass production of cotton-like products were yielded after firing at 1300 °C and 1400 °C (Figure 1c,d).

**Figure 2:**
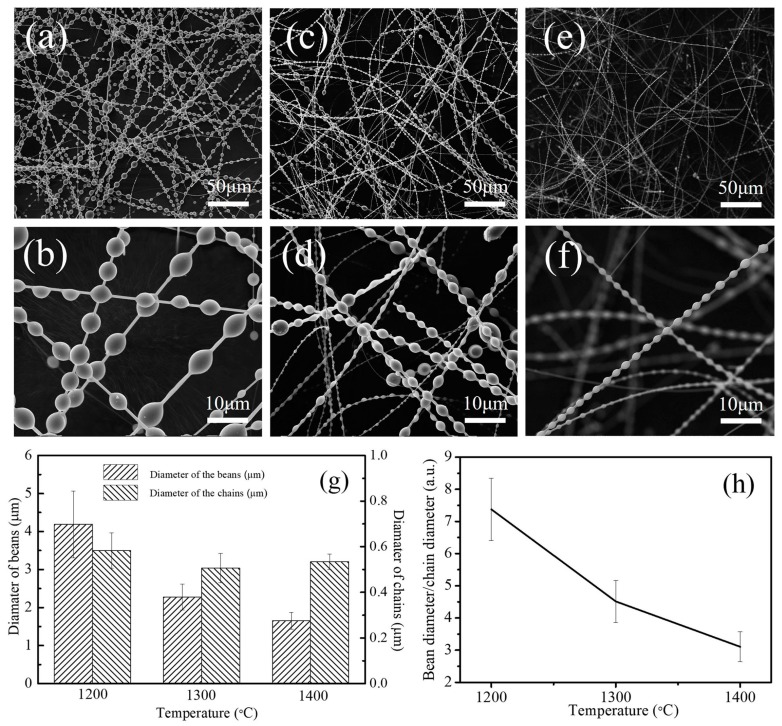
Scanning electron microcopy (SEM) images of the as-achieved products as a function of the firing temperature, (**a**,**b**) 1200 °C; (**c**,**d**) 1300 °C; (**e**,**f**) 1400 °C; (**g**) histogram for the diameter of the beans and chains in a necklace-like structure; (**h**) line chart representing the ratio of the beans’ diameter to the chains’ diameter.

The microstructures of the cotton-like products collected from the corundum crucible after firing at 1200 °C, 1300 °C, and 1400 °C were further observed by SEM and are presented in Figure 2. SEM images at low magnification (Figure 2a,c,e) revealed that the morphology of the as-prepared products was that of 1D fibers, and their length up to several millimeters. Higher-magnification images (Figure 2b,d–f) further indicated that the 1D fibers exhibited a necklace-like morphology. The beans wrapping on the fibers after firing at 1200 °C (Figure 2b) were inhomogeneous in size, and their diameters ranged from 3 to 6 μm. When increasing the temperature to 1300 °C, the average diameter of the beans reduced, while the diameters of the chains (Figure 2d) decreased slightly. As shown in Figure 2f, the products obtained at 1400 °C had a uniform morphology with periodical beads, and the average diameter of the beans and chains was 1.65 μm and 0.53 μm, respectively. The sizes of the beans and chains as well as their ratios are presented in Figure 2g,h. Clearly, the diameter of the beans decreased from about 4.2 μm to 1.8 μm, and the diameter of the chains slightly decreased, thus the ratio between the beans’ diameter and the chains’ diameter decreased from 8 to 3 when the temperature increased from 1200 °C to 1400 °C.

### 3.2. Components Analysis

**Figure 3:**
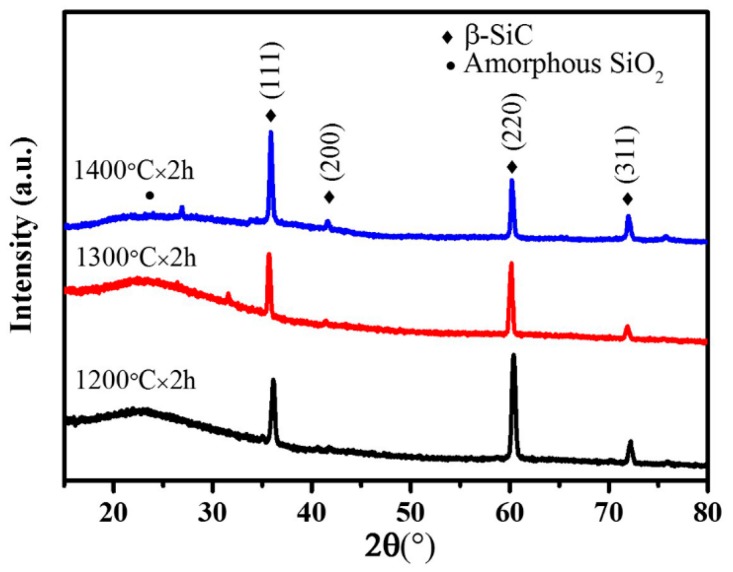
X-ray diffraction (XRD) patterns of the as-received samples.

The XRD patterns of the as-prepared products as a function of the firing temperature are shown in Figure 3. The diffraction peaks in the pattern shown in Figure 3 were assigned to (111), (200), (220), and (311) planes of cubic β-SiC (JCPDS card No. 29-1129); in addition, the β-SiC peak heights enhanced as the temperature increased. These sharp diffraction peaks further certified that the as-synthesized products were well crystallized. Apart from the β-SiC, a broad diffraction peak centered at about 23° was also observed, indicating the existence of an amorphous SiO_2_ phase. On increasing the temperature to 1400 °C, the amorphous SiO_2_ peak almost disappeared, but not completely.

**Figure 4:**
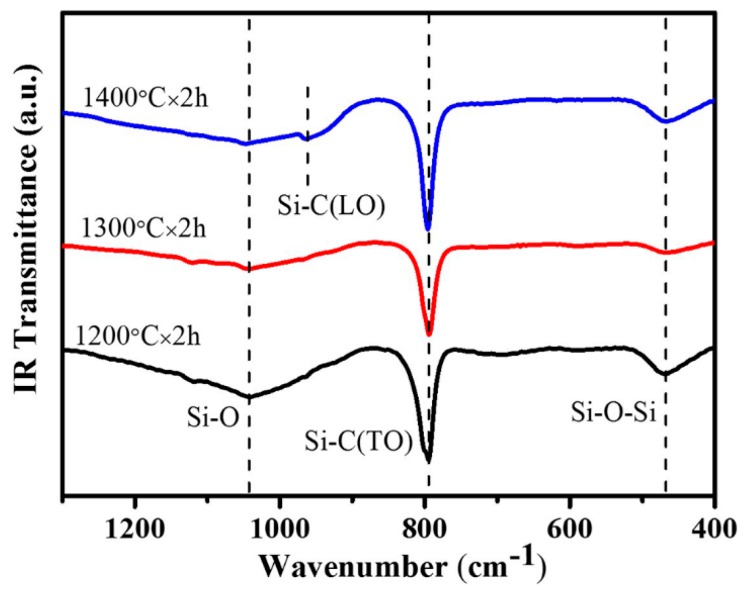
Fourier-transform-infrared spectroscopy (FT-IR) absorbance spectrum of the as-received samples.

Furthermore, FT-IR spectroscopy was used to analyze the composition of the the as-received samples. As indicated in Figure 4, three absorption peaks were detected from the fibers and could be assigned to a transversal optic (TO) Si–C stretching vibration at 794 cm^−1^ and a longitudinal optic (LO) Si–C stretching vibration at around 950 cm^−1^ [18,19]. Besides, the absorption peak at around 436 cm^−1^ was attributed to a Si–O–Si stretching vibration of amorphous SiO_2_ [20]. So, the results of FT-IR spectroscopy also testified the existence of SiC and SiO_2_.

**Figure 5:**
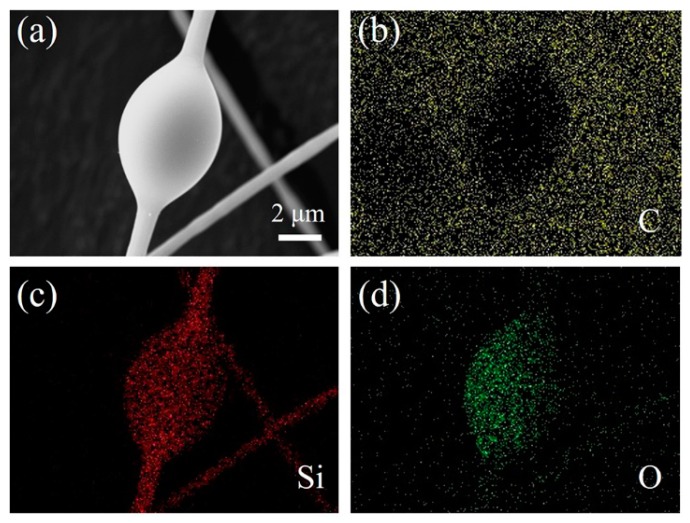
Mapping the energy dispersive X-ray spectroscopy (EDS) patterns of the SiC/SiO_2_ heterojunctions synthesized at 1200 °C: (**a**) SEM images of the products; (**b**) C element; (**c**) Si element; (**d**) O element.

An EDS analysis was further carried out to verify the elements in the products. The selected area and corresponding EDS patterns are illustrated in Figure 5. The necklace-like fibers were comprised of Si, C, and O elements; the content of C in the chains was much higher than in the beans, and the O content in the beans was much higher than in the nanochains. Therefore, the chains were mainly composed of SiC, and the beans maybe amorphous SiO_2_.

**Figure 6:**
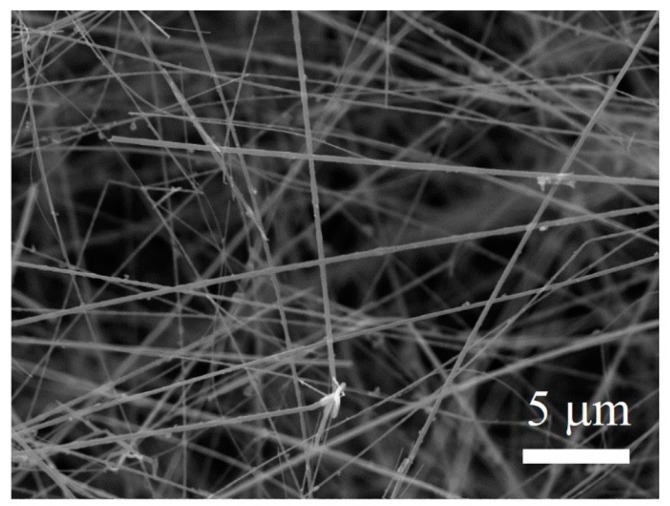
SEM images of the as-achieved products (firing at 1200 °C) after hydrofluoric acid (HF) treatment.

To further illuminate the chemical composition of the SiO_2_ bean in the nanochains, the as-prepared products were treated by a hydrofluoric acid (HF) solution for 12 h to remove SiO_2_. A SEM image of the as-received samples after HF treatment is presented in Figure 6. As shown in Figure 6, the specimens after HF treatment showed a different morphology. Obviously, the SiO_2_ beans in the nanochains almost disappeared, and the chains became pure SiC NWs. Together with the XRD, FT-IR, and EDS results, we could conclude that the microchains were composed of crystalline SiC nanochains and amorphous SiO_2_ beans. This conclusion was further verified by HRTEM.

### 3.3. Microstructure

**Figure 7:**
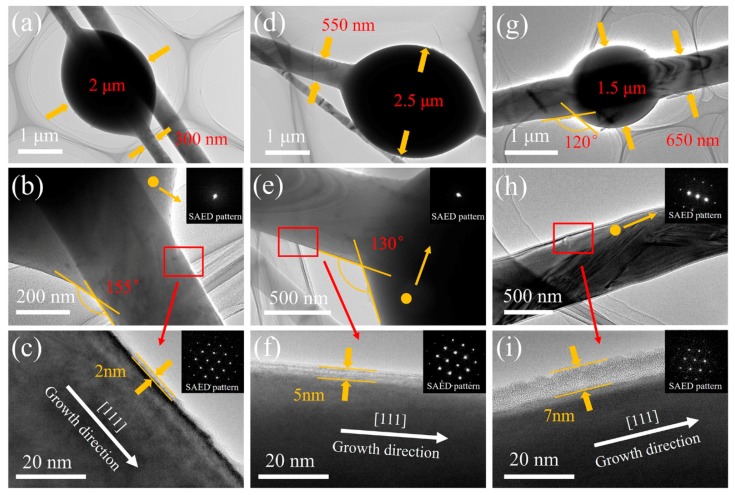
Typical transmission electron microscopy (TEM) images, high-resolution transmission electron microscopy (HRTEM) lattice images, and SAED patterns of the as-prepared samples: (**a**–**c**) 1200 °C; (**d**–**f**) 1300 °C; (**g**–**i**) 1400 °C.

The detailed morphologies and crystalline structures of the products were further characterized by TEM and HRTEM, as shown in Figure 7. As mentioned above, the necklace-like structure formed at different temperatures appeared to evolve with the temperature: in other words, the ratio of the beans’ diameter to the chains’ diameter varied with the firing temperatures. This was further confirmed, as shown in Figure 7a,d,g. As shown in Figure 7, we could observe that the diameter of the chain increased as the temperature increased. Later, the wetting angles also decreased from 155°, to 130°, and to 120° after firing at 1200 °C, 1300 °C, and 1400 °C, respectively, which could serve as an evidence of the formation of necklace-like SiC/SiO_2_ heterojunctions. The core-shell structure could be observed along the growth direction ([111] orientation) in HRTEM images (Figure 7c,f,i), and the thickness of the SiO_2_ shell increased on rising of the temperature increased, from 2 nm, to 3 nm, and to 7 nm, at 1200 °C, 1300 °C, and 1400 °C, respectively. Moreover, the selected area electron diffraction (SAED) patterns confirmed that the beans wrapping on the chains were amorphous, while the core of the chain possessed a good crystal structure, which matched well with the XRD results. The combination of the XRD, SEM, EDS, FT-IR, and HRTEM analyses of the as-prepared samples revealed the characteristics of the necklace-like SiC/SiO_2_ core-shell heterojunctions structure.

### 3.4. Photoluminescence

**Figure 8:**
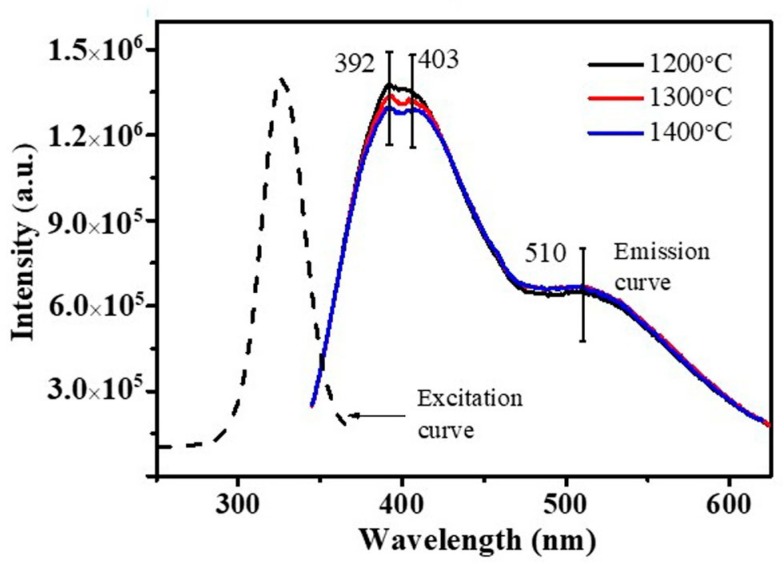
Emission spectra of SiC/SiO_2_ heterojunctions synthesized at different temperatures.

The morphologies of the SiC/SiO_2_ heterojunctions with beans of different sizes were tuned by adjusting the temperature, which indicated that these products could be a promising performance-tuning material for photoelectronic devices. Herein, the room temperature PL spectra of the as-synthesized products were measured at the excitation of 330 nm. As shown in Figure 8, the emission peaks ranging from around 392 nm (3.16 eV) to 403 nm (3.08 eV) were located in the violet–blue spectral range, which might be attributed to the defect-effect or the recombination of quantum confinement [21,22], such as stacking faults. Interestingly, the emission peak intensity reduced consistently with the increase in the firing temperature and decreased along with the increase of the SiO_2_ shell thickness. This further indicated that the amount and diameter of the SiO_2_ beans and the thickness of the SiO_2_ shell in the structure had a considerable impact on the PL properties. Meanwhile, the as-synthesized SiC/SiO_2_ heterojunctions exhibited a considerable blue shift in comparison with the SiC-SiO_2_ core-shell NWs described in a previous report [23], which might be attributed to a high concentration defect on the interface of β-SiC and SiO_2_.

**Figure 9:**
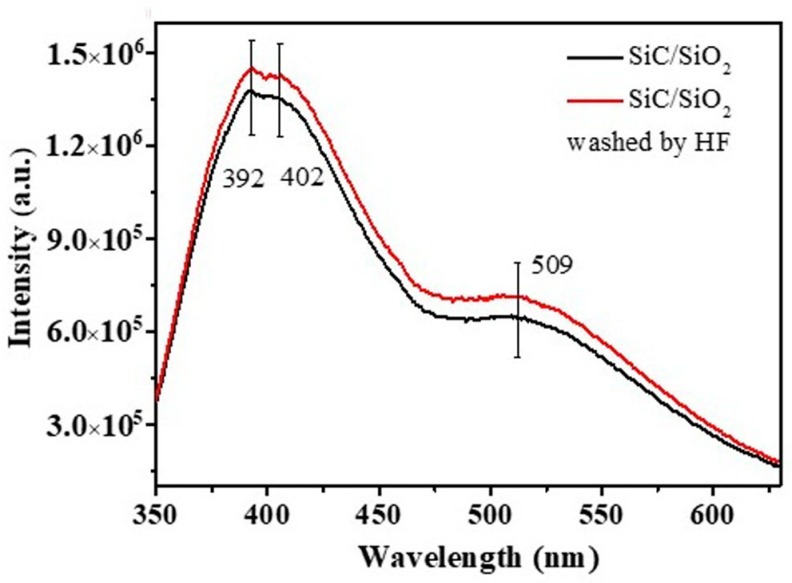
Emission spectra of SiC/SiO_2_ heterojunctions after HF treatment.

Figure 9 shows the PL spectra of the as-synthesized products after treating with the HF solution, revealing the influence of the SiO_2_ shell and bean structure on the PL properties at room temperature. As shown, the intensity of the emission peak was enhanced by 20% in comparison to the samples not treated with HF, and the emission curve could be simulated and divided into three unimodal curves centered at 392 nm, 402 nm, and 509 nm, respectively. This proved that the increase of PL intensity was due to the decreasing SiO_2_ shell thickness, which was consistent with previous results [24]. Low SiO_2_ shell thickness was promoted the PL properties of the SiC/SiO_2_ heterojunctions. On the basis of the aforementioned results, these emission peaks could be attributed to a defective luminescence mechanism.

As for the luminescence mechanism of the SiC fibers, it is still not completely understood yet. Nevertheless, previous reports indicated that the PL properties of SiC NWs strongly depend on the the microstructure and morphology of SiC NWs. For instance, Wei et al. [3] reported that the PL peak at 391 nm was attributed to the hexagonal shape and abundant structure defects in the SiC NWs’ core, such as stacking faults, twins, and polytypic growth. Nishikawa et al. [25] attributed the emission peak at 470 nm to the neutral oxygen vacancy (≡Si–Si≡), while the 412 nm band corresponded to some intrinsic diamagnetic defect center, such as the twofold coordinated silicon lone pair centers (O–Si–O). Wang et al. [26] ascribed the blue shift of the PL to the morphology and oxygen vacancy. For instance, the hexagonal prism-shaped SiC nanostructures exhibit emission peaks at 470 nm and around 480–600 nm [27], whereas the tubular β-SiC NWs have two PL emission peaks at 468 nm and 570 nm [28], respectively. These results reveal that structure, morphology, and defects have a great influence on the emission peaks of SiC nanostructures. Hence, the PL spectrum mainly results from the defect effect, the recombination of quantum confinement in the SiO_2_ beans, or in the presence of irregularities on the interface between the SiO_2_ shell and the SiC core, such as oxygen vacancy or twins. Nevertheless, how the structure defects and other factors affect the PL properties still needs to be further studied.

### 4.5. Growth Mechanism

The growth mechanism of SiC/SiO_2_ heterostructures has been described in many works. For instance, Wu et al. [29] proposed that the surface deposition sites on the surface of SiC NWs play a critical role in the formation of SiC/SiO_2_ heterostructures. Hou et al. [14] suggested that the vapor-solid (VS) growth mechanism of SiC/SiO_2_ nanochains heterojunctions are generated by the modulation process and that the growth of SiO_2_ beads is controlled by gaseous SiO. The VS growth mechanism better explains the growth of SiC/SiO_2_ heterojunctions in this work, because of the absence of a catalyst. However, the formation of periodic beans indicated that the growth process is not merely controlled by the VS mechanism. Furthermore, the SiO_2_ shell can melt, flow along the SiC NWs, and cover their surfaces at high temperatures [3]. Then, liquid SiO_2_ can be completely separated into spheres and form the necklace-like structures because of Rayleigh instability and poor wettability between SiC and SiO_2_.

**Figure 10:**
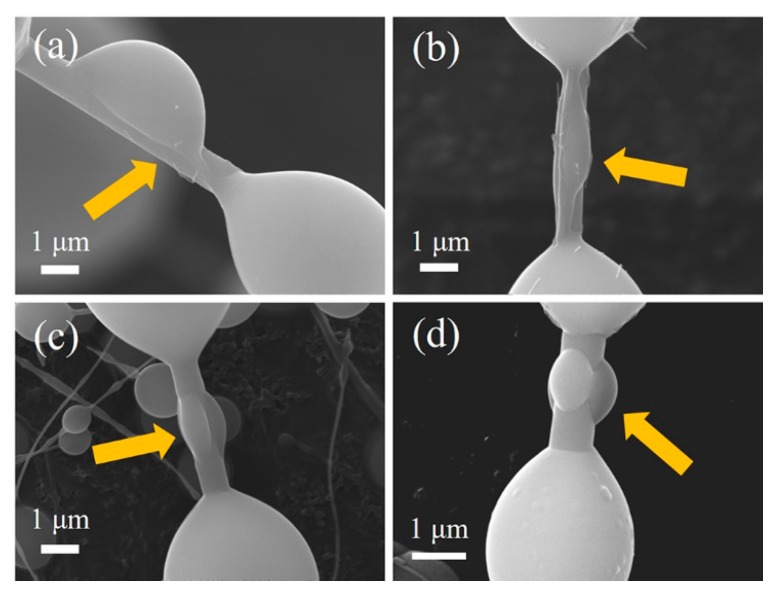
SEM images of the liquid SiO_2_ movement of the as-prepared samples after firing at 1400 °C. (**a**) SiO_2_ bean; (**b**) Migration of SiO_2_ droplet; (**c**) shrinkage of SiO_2_ droplet; (**d**) SiO_2_ bean

As mentioned in our previous work [17], the growth mechanism of the as-synthesized SiC/SiO_2_ core-shell nanochains is most likely to be controlled by the combination of the VS growth and the modulation procedures. The introduction of molten salt and firing in air (embedding in graphite powders) facilitated the SiO vapor to easily reach a supersaturated state and deposited directly on the surface of the SiC chains during in the cooling process [30]. The formation of the SiO_2_ beans could be mainly attributed to Rayleigh instability and surface tension. The movement of the SiO_2_ beans was observed in this work, as shown in Figure 10. First of all, bean adhesion to the SiC NWs was unstable (Figure 10a), hence liquid SiO_2_ could migrate along the SiC NWs (in Figure 10b). After the formation of a SiO_2_ liquid tricklet, the SiC NWs were still covered by residual liquid SiO_2_, which tended to shrink and adopt a spherical shape as a result of the surface tension (Figure 10c,d). In the modulation process, upon temperature increase, the viscosity of the liquid SiO_2_ decreased, while its flowability increased. Thus, the liquid SiO_2_ shrunk quickly and could be separated into several segments, which formed the SiO_2_ beans because of the poor wettability of the SiC–SiO_2_ interface due to surface energy and Rayleigh instability, thereby decreasing the size of the SiO_2_ beans and generating different sizes of beans and nanochains, as demonstrated by the SEM (Figure 2) and HRTEM analyses (Figure 7). The SiO_2_ beads formed in the cooling stage, and the bead shape could be tuned by modulating the soaking time [16]. On the other hand, amorphous SiO_2_ could be generated by the oxidation of the SiC NWs (SiC + CO → SiO_2_ + 3C (g)), resulting in the formation of a SiC/SiO_2_ core-shell structure. The thickness of the SiO_2_ shell on the nanochains tended to increase as the temperature increased from 1200 °C to 1400 °C (in Figure 7). Thus, the bean sizes and the thickness of the SiO_2_ shell in the necklace-like SiC/SiO_2_ heterostructures were tuned by adjusting the temperature, and their growth mechanism involved both a VS growth mechanism and modulation procedure via a molten salt-mediated process.

## 4. Conclusions

(1) A mass production of necklace-like SiC/SiO_2_ heterojunctions was successfully achieved by a molten salt-mediated chemical vapor reaction technique, without a catalyst and a flowing gas. Silicon powder and graphite powder were used as material precursors, and sodium chloride and sodium fluoride were adopted as molten salts.
(2) The as-prepared products synthesized above 1200 °C consisted of fibers and of beans wrapped around the fibers. The fibers were about several millimeters in length, while the diameter of the beans was around several micrometers and tended to decrease as the temperature increased.
(3) The results of the XRD, EDS, FT-IR, and TEM analyses confirmed that the chains were composed of a crystalline SiC core and an amorphous SiO_2_ shell, and the beans were composed of amorphous SiO_2_. Meanwhile, the growth direction of SiC cores was perpendicular to the (111) plane.
(4) The bean sizes and thickness of the SiO_2_ shell in the as-prepared necklace-like SiC/SiO_2_ heterostructures were tuned by adjusting the reaction temperature, that is, by increasing temperature, the average size of the beans decreased from 4 μm to 1μm, while the thickness of the SiO_2_ shell increased from 2 nm to 7 nm. The products exhibited a brilliant photoluminescence at 392 nm (3.16 eV), which suggests that they are a promising material for optoelectronic applications.
(5) The growth mechanism leading to the formation of the necklace-like structure involved a molten salt-mediated process, the VS growth mechanism of the SiC/SiO_2_ core-shell fibers, and the modulation process. Furthermore, Rayleigh instability and surface tension were supposed to be the driving forces for the migration and modulation process of the necklace-like structure.
